# Chloridodimeth­yl(thio­semi­carbazide)tin(IV) chloride

**DOI:** 10.1107/S1600536810038705

**Published:** 2010-10-02

**Authors:** Laura Cortes C., Ana E. Burgos C., Coco K. Y. A. Okio

**Affiliations:** a Departamento de Quimíca, Universidad Nacional de Colombia, Sede Bogotá, Bogotá, Colombia

## Abstract

In the title salt, [Sn(CH_3_)_2_Cl(CH_4_N_3_S)]Cl, the Sn^IV^ atom is five-coordinated in a distorted trigonal-bipyramidal geometry with two methyl groups and one S atom in the equatorial plane, and one N atom and one Cl atom occupying the apical positions. In the crystal, mol­ecules are linked by inter­molecular N—H⋯S hydrogen bonds with set graph-motif *C*(4) along [010]. N—H⋯ Cl hydrogen bonds with graph-set motif *D*(2) and *D*_3_^3^(10) link cations and anions.

## Related literature

For a related structure, see: Delgado *et al.* (2009 ▶). For graph-set motifs, see: Bernstein *et al.* (1995 ▶). For the biological activity of organotin(IV) complexes, see: Davies & Smith (1982 ▶).
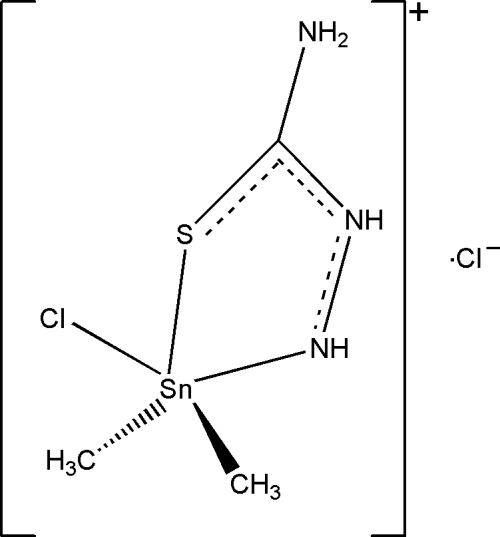


## Experimental

### 

#### Crystal data

[Sn(CH_3_)_2_Cl(CH_4_N_3_S)]Cl
*M* *_r_* = 309.79
Monoclinic, 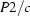

*a* = 13.4980 (12) Å
*b* = 6.2470 (5) Å
*c* = 12.7160 (13) Å
β = 108.871 (10)°
*V* = 1014.60 (16) Å^3^
*Z* = 4
Mo *K*α radiation
μ = 3.19 mm^−1^
*T* = 293 K
0.13 × 0.10 × 0.09 mm

#### Data collection

Nonius KappaCCD diffractometer
Absorption correction: multi-scan (*SORTAV*; Blessing, 1995 ▶) *T*_min_ = 0.613, *T*_max_ = 0.809
4452 measured reflections
2915 independent reflections
2475 reflections with *I* > 2σ(*I*)
*R*_int_ = 0.020

#### Refinement

*R*[*F* ^2^ > 2σ(*F* ^2^)] = 0.037
*wR*(*F* ^2^) = 0.103
*S* = 1.14
2915 reflections
91 parameters
H-atom parameters constrained
Δρ_max_ = 1.04 e Å^−3^
Δρ_min_ = −1.52 e Å^−3^

### 

Data collection: *COLLECT* (Nonius, 1998 ▶); cell refinement: *DENZO* (Otwinowski & Minor, 1997 ▶); data reduction: *DENZO*; program(s) used to solve structure: *SHELXS97* (Sheldrick, 2008 ▶); program(s) used to refine structure: *SHELXL97* (Sheldrick, 2008 ▶); molecular graphics: *PLATON* (Spek, 2009 ▶); software used to prepare material for publication: *SHELXL97*.

## Supplementary Material

Crystal structure: contains datablocks I, global. DOI: 10.1107/S1600536810038705/bx2306sup1.cif

Structure factors: contains datablocks I. DOI: 10.1107/S1600536810038705/bx2306Isup2.hkl

Additional supplementary materials:  crystallographic information; 3D view; checkCIF report

**Fig. 1.**
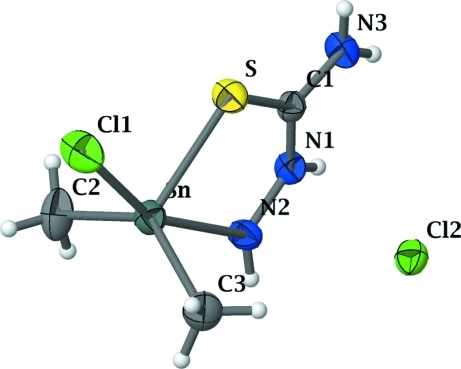
The molecular structure of (I), with the atom-numbering scheme. Displacement ellipsoids are drawn at the 50% probability level. H atoms have been omitted.

Enhanced figure: interactive version of Fig. 1

## Figures and Tables

**Table 1:** Hydrogen-bond geometry (Å, °)

*D*—H⋯*A*	*D*—H	H⋯*A*	*D*⋯*A*	*D*—H⋯*A*
N1—H1⋯Cl2^i^	0.86	2.3555	3.147 (4)	153.17
N2—H2⋯S^ii^	0.86	2.5549	3.327 (3)	149.90

Symmetry codes: (i) 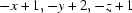; (ii) 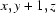.

**Fig. 2.**
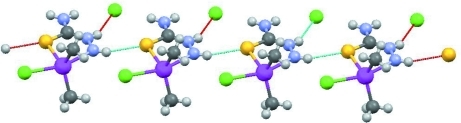
Unit-cell packing diagram for (I). Hydrogen bonds are shown as dashed lines.

## supplementary crystallographic information

### Comment

Organotin(IV) complexes have been extensively studied due to the diversity of structures that such compounds can form and to their potential biological activities as well as their wide industrial and agricultural applications (Davies & Smith, 1982). In the framework of our research for new organotin(IV) compounds (Delgado *et al.*, 2009), we report here the crystal structure of the title compound (I). The asymmetric unit is formed by one cation and one anion. The Sn atom is five-coordinate in a distorted trigonal–bipyramidal geometry. The distorted trigonal-bipyramidal coordination polyhedron has two methyl groups and one S atom in the equatorial plane, the N2 and Cl1 atom occupying the apical positions. In the crystal, molecules are linked by intermolecular N—H···S hydrogen bonds with set graph-motif C(4) along [010]. N—H··· Cl hydrogen bond linking cations and anions with set graph-motif D(2) and D_3_^3^(10) , Table 1 and Fig.2. The C1-S and C1-N1 bond distances are quite shorter than the ones reported for C-S and C-N single bonds (1.755 (4), 1.366 (6)) (Delgado *et al.*, 2009), suggesting the delocalization of the C=S double bond on the SCN moiety.

### Experimental

Compound (I) was obtained by reacting dimethyltin (IV) dichloride (220 mg, 1 mmol) with thiosemicarbazide (68 mg, 0.75 mmol) in methanol under reflux for 3 h. Colourless crystals suitable for X-ray analysis were grown by slow solvent evaporation.

### Refinement

H atoms were positioned geometrically, with C—H, N—H distances of 0.96 and 0.86Å respectively, and constrained to ride on their parent atoms, with *U*_iso_(H) = 1.5U_eq_(C) and 1.2U_eq_(N)

### Crystal data

**Table:**

[Sn(CH_3_)_2_Cl(CH_4_N_3_S)]Cl	*F*(000) = 596
*M**_r_* = 309.79	*D*_x_ = 2.028 Mg m^−^^3^
Monoclinic, *P*2/*c*	Mo *K*α radiation, λ = 0.71073 Å
Hall symbol: -P 2yc	Cell parameters from 1915 reflections
*a* = 13.4980 (12) Å	θ = 1.0–30.0°
*b* = 6.2470 (5) Å	µ = 3.19 mm^−^^1^
*c* = 12.7160 (13) Å	*T* = 293 K
β = 108.871 (10)°	Prism, colorless
*V* = 1014.60 (16) Å^3^	0.13 × 0.10 × 0.09 mm
*Z* = 4	

### Data collection

**Table:**

Nonius KappaCCD diffractometer	2915 independent reflections
Radiation source: fine-focus sealed tube	2475 reflections with *I* > 2σ(*I*)
graphite	*R*_int_ = 0.020
π scans	θ_max_ = 30.0°, θ_min_ = 1.6°
Absorption correction: multi-scan (*SORTAV*; Blessing, 1995)	*h* = −18→18
*T*_min_ = 0.613, *T*_max_ = 0.809	*k* = −7→8
4452 measured reflections	*l* = −17→17

### Refinement

**Table:**

Refinement on *F*^2^	Primary atom site location: structure-invariant direct methods
Least-squares matrix: full	Secondary atom site location: difference Fourier map
*R*[*F*^2^ > 2σ(*F*^2^)] = 0.037	Hydrogen site location: inferred from neighbouring sites
*wR*(*F*^2^) = 0.103	H-atom parameters constrained
*S* = 1.14	*w* = 1/[σ^2^(*F*_o_^2^) + (0.0482*P*)^2^ + 1.1794*P*] where *P* = (*F*_o_^2^ + 2*F*_c_^2^)/3
2915 reflections	(Δ/σ)_max_ = 0.001
91 parameters	Δρ_max_ = 1.04 e Å^−^^3^
0 restraints	Δρ_min_ = −1.52 e Å^−^^3^

### Special details

**Table:**

Experimental. Absorption correction: multi-scan from symmetry-related measurements (SORTAV; Blessing, 1995)
Geometry. All e.s.d.'s (except the e.s.d. in the dihedral angle between two l.s. planes) are estimated using the full covariance matrix. The cell e.s.d.'s are taken into account individually in the estimation of e.s.d.'s in distances, angles and torsion angles; correlations between e.s.d.'s in cell parameters are only used when they are defined by crystal symmetry. An approximate (isotropic) treatment of cell e.s.d.'s is used for estimating e.s.d.'s involving l.s. planes.
Refinement. Refinement of *F*^2^ against ALL reflections. The weighted *R*-factor *wR* and goodness of fit *S* are based on *F*^2^, conventional *R*-factors *R* are based on *F*, with *F* set to zero for negative *F*^2^. The threshold expression of *F*^2^ > σ(*F*^2^) is used only for calculating *R*-factors(gt) *etc*. and is not relevant to the choice of reflections for refinement. *R*-factors based on *F*^2^ are statistically about twice as large as those based on *F*, and *R*- factors based on ALL data will be even larger.

### Fractional atomic coordinates and isotropic or equivalent isotropic displacement parameters (Å^2^)

**Table:**

	*x*	*y*	*z*	*U*_iso_*/*U*_eq_	
Sn	0.18008 (2)	0.64590 (4)	0.54686 (2)	0.03327 (10)	
Cl1	0.10951 (9)	0.27486 (19)	0.52290 (13)	0.0570 (3)	
Cl2	0.37205 (8)	0.89418 (16)	0.34229 (9)	0.0384 (2)	
S	0.34762 (8)	0.45496 (15)	0.57559 (10)	0.0394 (2)	
N1	0.4153 (3)	0.8592 (5)	0.6156 (3)	0.0304 (6)	
H1	0.4647	0.9508	0.6420	0.036*	
N2	0.3105 (2)	0.9290 (5)	0.5800 (3)	0.0348 (7)	
H2	0.2937	1.0621	0.5707	0.042*	
N3	0.5392 (3)	0.6073 (5)	0.6316 (3)	0.0371 (7)	
H3AN	0.5859	0.7063	0.6494	0.045*	
H3BN	0.5575	0.4762	0.6282	0.045*	
C1	0.4390 (3)	0.6566 (6)	0.6093 (3)	0.0291 (7)	
C2	0.1509 (4)	0.7230 (10)	0.6955 (4)	0.0548 (12)	
H2A	0.1548	0.8753	0.7060	0.082*	
H2B	0.2022	0.6549	0.7569	0.082*	
H2C	0.0823	0.6738	0.6913	0.082*	
C3	0.1119 (4)	0.7551 (9)	0.3820 (4)	0.0471 (10)	
H3A	0.0640	0.8695	0.3806	0.071*	
H3B	0.0749	0.6396	0.3362	0.071*	
H3C	0.1658	0.8060	0.3542	0.071*	

### Atomic displacement parameters (Å^2^)

**Table:**

	*U*^11^	*U*^22^	*U*^33^	*U*^12^	*U*^13^	*U*^23^
Sn	0.03237 (15)	0.03147 (15)	0.03651 (16)	−0.00300 (10)	0.01187 (11)	−0.00248 (10)
Cl1	0.0403 (5)	0.0344 (5)	0.0951 (9)	−0.0086 (5)	0.0201 (6)	−0.0063 (6)
Cl2	0.0369 (5)	0.0377 (5)	0.0413 (5)	−0.0039 (4)	0.0138 (4)	0.0036 (4)
S	0.0352 (5)	0.0245 (4)	0.0595 (6)	−0.0027 (4)	0.0168 (4)	−0.0017 (4)
N1	0.0299 (15)	0.0266 (14)	0.0342 (15)	−0.0046 (12)	0.0097 (12)	−0.0039 (12)
N2	0.0285 (15)	0.0259 (14)	0.0494 (19)	0.0002 (13)	0.0117 (13)	0.0017 (14)
N3	0.0316 (16)	0.0367 (17)	0.0444 (19)	−0.0017 (14)	0.0141 (14)	−0.0064 (14)
C1	0.0322 (18)	0.0308 (17)	0.0262 (15)	0.0006 (14)	0.0121 (13)	0.0015 (13)
C2	0.062 (3)	0.067 (3)	0.046 (3)	−0.017 (3)	0.031 (2)	−0.010 (2)
C3	0.042 (2)	0.057 (3)	0.038 (2)	−0.002 (2)	0.0072 (17)	0.002 (2)

### Geometric parameters (Å, °)

**Table:**

Sn—C2	2.107 (4)	N3—C1	1.325 (5)
Sn—C3	2.112 (4)	N3—H3AN	0.8600
Sn—N2	2.434 (3)	N3—H3BN	0.8600
Sn—S	2.4771 (11)	C2—H2A	0.9600
Sn—Cl1	2.4870 (12)	C2—H2B	0.9600
S—C1	1.718 (4)	C2—H2C	0.9600
N1—C1	1.315 (5)	C3—H3A	0.9600
N1—N2	1.408 (4)	C3—H3B	0.9600
N1—H1	0.8600	C3—H3C	0.9600
N2—H2	0.8600		
			
C2—Sn—C3	132.0 (2)	C1—N3—H3BN	120.0
C2—Sn—N2	90.75 (17)	H3AN—N3—H3BN	120.0
C3—Sn—N2	89.75 (16)	N1—C1—N3	117.6 (3)
C2—Sn—S	113.85 (17)	N1—C1—S	123.5 (3)
C3—Sn—S	112.70 (14)	N3—C1—S	118.9 (3)
N2—Sn—S	75.51 (8)	Sn—C2—H2A	109.5
C2—Sn—Cl1	98.48 (16)	Sn—C2—H2B	109.5
C3—Sn—Cl1	98.80 (15)	H2A—C2—H2B	109.5
N2—Sn—Cl1	157.72 (8)	Sn—C2—H2C	109.5
S—Sn—Cl1	82.21 (4)	H2A—C2—H2C	109.5
C1—S—Sn	103.38 (13)	H2B—C2—H2C	109.5
C1—N1—N2	121.2 (3)	Sn—C3—H3A	109.5
C1—N1—H1	119.4	Sn—C3—H3B	109.5
N2—N1—H1	119.4	H3A—C3—H3B	109.5
N1—N2—Sn	115.2 (2)	Sn—C3—H3C	109.5
N1—N2—H2	122.4	H3A—C3—H3C	109.5
Sn—N2—H2	122.4	H3B—C3—H3C	109.5
C1—N3—H3AN	120.0		
			
C2—Sn—S—C1	−79.8 (2)	S—Sn—N2—N1	−9.3 (2)
C3—Sn—S—C1	88.0 (2)	Cl1—Sn—N2—N1	−9.7 (4)
N2—Sn—S—C1	4.48 (15)	N2—N1—C1—N3	172.0 (3)
Cl1—Sn—S—C1	−175.69 (14)	N2—N1—C1—S	−8.9 (5)
C1—N1—N2—Sn	13.1 (4)	Sn—S—C1—N1	0.0 (4)
C2—Sn—N2—N1	105.2 (3)	Sn—S—C1—N3	179.2 (3)
C3—Sn—N2—N1	−122.9 (3)		

### Hydrogen-bond geometry (Å, °)

**Table:**

*D*—H···*A*	*D*—H	H···*A*	*D*···*A*	*D*—H···*A*
N1—H1···Cl2^i^	0.86	2.3555	3.147 (4)	153.17
N2—H2···S^ii^	0.86	2.5549	3.327 (3)	149.90

Symmetry codes: (i) −*x*+1, −*y*+2, −*z*+1; (ii) *x*, *y*+1, *z*.
